# Flame-Synthesis of Carbon Nanotube Forests on Metal Mesh Structure: Dependence, Morphology, and Application

**DOI:** 10.3390/nano9091188

**Published:** 2019-08-22

**Authors:** Xuhai Xiong, Pu Zhao, Rong Ren, Xu Cui, Shude Ji

**Affiliations:** 1 Liaoning Key Laboratory of Advanced Polymer Matrix Composites, Shenyang Aerospace University, Shenyang 110136, China; 2 Shenyang National Laboratory for Materials Science, Advanced Carbon Division, Institute of Metal Research, Chinese Academy of Sciences, Shenyang 110016, China

**Keywords:** MWCNT forests, flame, growth mechanism, turbulent flow simulation, thermoplastic composite welding

## Abstract

Multi-walled carbon nanotubes (MWCNTs) in the form of “forests” were synthesized directly on the surface of stainless steel (SS) mesh from ethanol flame volume. The growth dependence of the MWCNT forests on the porosity of SS mesh substrate and the morphologies and growth mechanism of the MWCNT forests were investigated in detail by a combination of turbulent flow simulation, scanning electron microscopy (SEM), transmission electron microscope (TEM), and Raman and X-ray diffraction (XRD) spectroscopy. The growth height of the MWCNT forests exhibited a strong dependence on the flame gas flow rate controlled by the porosity of SS mesh substrate, and the maximum averaged height of the MWCNT forests reached 34 μm. Most MWCNTs grew perpendicularly on the surface of SS wires, and some branch, welded, and spiral structures were observed by SEM and TEM. The MWCNT-decorated mesh was used as a novel heating element to weld glass-fabric-reinforced polyetherimide (GF/PEI) thermoplastics. We found that the maximum tensile lap-shear strength (LSS) of the welded joints could reach 39.21 MPa, an increase of 41% in comparison with that of conventional SS mesh-based joints.

## 1. Introduction

Carbon nanotube (CNT) forests are grown from adjacent, short-term, continuously accumulated CNTs at crowded catalyst sites by “crowding effects” into a collectively ordered structure, and are also known as “VACNTs” or “arrays” or “carpet” due to their vertical and aligned morphology [1,2,3,4]. They possess high collimation, high purity, and high specific surface area, and exhibit superior mechanical properties, electrical conductivity, and heat transfer properties [5,6]. The unique structure and properties of CNT forests have opened up many new and important applications, covering a wide range of fields from field emitters [7], touchpads [8], planar incandescent light sources [9], flexible charge collectors [10], high-power and high-density supercapacitors [11,12], and dry adhesives [13] to oil–water separators [14,15,16].

Resistance welding is an important fusion joining technology for thermoplastic composites and has been widely used in aerospace parts [17,18]. A conductive stainless steel (SS) mesh is required to be inserted into the welding region as in situ heating element. However, the intra-layer interface debonding of the implant is the main failure mechanism for the welded joints. CNTs and VACNTs have been investigated widely as a nano-reinforcement to enhance the interfacial bond strength between fiber and resin or the interlaminar bond strength of composite laminates [19,20,21,22,23,24]. It is expected that if the surface of the SS mesh, which is the most widely-used heating element for the resistance welding of the thermoplastic composites, is modified by CNT forests, it will be beneficial to the strength of the resistance welded joint.

The chemical vapor deposition (CVD) method combined with template processing technology has been widely used to prepare CNT forests [25,26,27], including thermal CVD, floating catalytic CVD, plasma enhanced CVD, alcohol catalytic or water assisted CVD [28], etc. It is not easy or economically feasible for the CVD method to prepare CNT forests at large scales because of the long reaction time, complex reaction equipment, and other variable factors. The development of controllable, economical, and highly tunable synthesis techniques is crucial for the practical application of CNT forests. Compared to the CVD method, flame synthesis is a low-cost and low-consumption method that does not require large and expensive equipment, fascinatingly, and the flame volume can simultaneously provide a carbon-rich chemical reaction environment and the heat requirement for CNT growth [29]. This one-step synthesis of carbon nanostructures in a short residence time has been proven to be a high-efficiency technique for continuous and high-volume production of CNTs [30,31]. Some researchers have prepared CNT forests using flame volume with the aid of an external electric or magnetic field [32,33,34,35]. However, there has been no well-known report on the direct synthesis of CNT forests by flame volume excluding a physical field. When a porous substrate is immersed into the flame volume, the gas carbon resource will form a directional flow, which is beneficial for the oriented growth of CNTs. This novel flame method based on a porous substrate is expected to be a simple, efficient, and promising technology by which to synthesize CNT forests as an alternative to the CVD method. 

In this paper, we first focused on the influencing factors of the flame growth of multi-walled CNT (MWCNT) forests on porous metal substrates. The turbulent *k-ε* fluid model was used to simulate the growth flame environment of MWCNT forests to obtain the optimum carbon feeding, and the concentration distribution of the carbon source on the surface of a single metal wire has been discussed. The predicted results were verified respectively by the flame growth experiments of MWCNT forests in term of their average growth height and distribution on metal wire. Microstructure, morphology, and growth mechanisms were characterized and elucidated. Lastly, the effects of flame-grown MWCNT forests on the improvement of the welded joint strength of thermoplastic composites was also studied.

## 2. Experimental Procedures

### 2.1. Materials and Reagents

The 304 stainless steel (SS) mesh was manufactured by Tengde Metal Wire Mesh Products Co. LTD, Hebei, China. Ni(NO_3_)_2_·6H_2_O was obtained from Shenyang Xinghua reagent factory, Shenyang, China. Commercially available EW140 orthogonal glass fabric was provided by Geshang Composite Materials Co. LTD, Zhejiang, China. Polyetherimide (PEI) was supplied by Dongguan Zhuyou Plastics Co. LTD, Dongguan, China. *N,N*-dimethylacetamide was provided by Shanghai Jinshan Jingwei Chemical Co. LTD, Shanghai, China. All reagents were of analytically pure grade and were used as received. 

### 2.2. MWCNT Forest Synthesis

The growth process of MWCNT forests was shielded by a long cylindrical quartz cavity to ensure the stable combustion of the ethanol flame. The 304 stainless steel mesh was used as the porous metal substrate for the growth of MWCNT forests, and Ni(NO_3_)_2_·6H_2_O was used as a catalyst. Ethanol fuel was the analytical reagent. 

**Figure 1:**
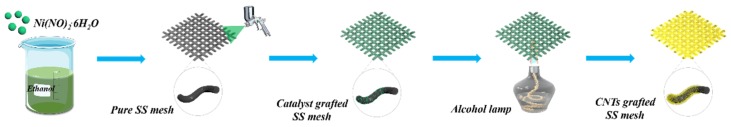
Experimental apparatus for carbon nanotube (CNT) flame growth.

The specific experimental steps for the growth of MWCNT forests were as follows (Figure 1): (1) Metal meshes with different specifications (Table 1) were cut into 20 mm × 20 mm uniform sizes. Subsequently, the as-obtained metal mesh patches were ultrasonically cleaned in acetone for 30 min. (2) A 1 mol/L Ni(NO_3_)_2_ ethanol solution was uniformly sprayed on the surface of the porous metal substrates by a spray gun, followed by drying in a 60 °C oven for 30 min. (3) The porous metal substrates with Ni(NO_3_)_2_ catalyst were positioned at 1/3 height of the strongly burning flames and kept for different times. (4) The MWCNT-grafted SS mesh was sandwiched by two PEI films, and this implant with a three-layer structure was then embedded into the single lap joint of two glass-fabric-reinforced polyetherimide (GF/PEI) laminates under a uniform initial pressure of 0.2 MPa. The input voltage and current were 20 V and 12 A, respectively, for the resistance welding.

### 2.3. Characterization

Microtopography of the MWCNT forests was observed by a Hitachi SU3500 SEM (Hitachi Hitachi Ltd., Tokyo, Japan), and their height was quantitatively measured based on SEM images. A high-resolution transmission electron microscope (FEI Tecnai G2 F20 S-Twin, FEI Inc., Hillsboro, USA) was used to characterize the micro-structural parameters of the single MWCNT. Raman spectroscope (Labram HR 800, Horiba Jobin Yvon Ltd., Paris, France) was performed to characterize the chemical structure of MWCNT forests. The crystal phases of samples were measured by X-ray diffraction (XRD-7000, Shimadzu Ltd., Kyoto, Japan) using Cu K_α_ radiation (λ = 0.15418 nm) with a scope of 5–90°. A handheld infrared thermal imager (Fluke Ti401 PRO, Fluke Ltd., Everett, USA) was used to test the temperature distribution of the ethanol flame. The single lap shear strength (LSS) of the welded specimens was tested using a universal testing machine (Instron 8801, Instron Inc., Boston, USA).

### 2.4. Computational Fluid Dynamics

This paper used ANSYS Fluent software (version 19.0, ANSYS Inc., Pittsburgh, USA) to simulate the vapor flow field of an ethanol flame. We first established a set of differential equations that control the flow. This set of governing equations included a continuity equation that reflected the conservation of fluid mass and an equation of motion that reflected the conservation of fluid momentum. The governing equations included the energy equation for the conservation of fluid energy. 

According to its structure, the unstructured non-uniform mesh was used to mesh the combustion chamber. The total number of grids was 135,548, which was able to meet the requirements of calculation accuracy. The whole simulation process was simplified to a two-dimensional flow around a circular cylinder model; that is, the gas flow passed through the gaps among parallel-arrayed circular cylinders with different spans. The upward flow rate of the carbon source gas formed by the ethanol flame is 0.5–1 m/s [36]. In this simulation process, 1m/s was taken as the initial flow rate to observe a more noticeable flow trend.

## 3. Results and Discussion

### 3.1. Growth and Characterization of MWCNT Forests

A non-porous thin metal sheet coated by catalyst was used as substrate for CNT growth in the traditional flame process [37,38,39]. The resultant CNTs were chaotic and their length exhibited weak uniformity due to the unstable flow and poor distribution of the carbon source feeding. The porous metal substrate was able to keep the flame volume steadily flowing, and thus more uniform velocity, temperature, and pressure fields were obtained. This was expected to facilitate the production of MWCNT forests with higher quality. Obviously, it is important to clearly understand the carbon gas–fluid state during the CNT growth process. A simplified *k-ε* turbulent fluid model was used to simulate the process to obtain the optimal carbon source feeding. 

**Figure 2:**
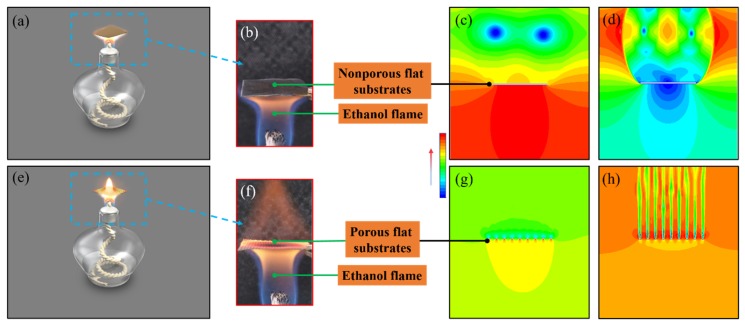
(**a**) Schematic diagram of flame on non-porous flat substrate; (**b**) real photo of flame on non-porous flat substrate; (**c**) pressure image and (**d**) flow velocity image for CNT growth on non-porous flat substrate; (**e**) schematic diagram of flame on porous flat substrate; (**f**) real photo of flame on porous flat substrate; (**g**) pressure image and (**h**) flow velocity image for CNT growth on the porous flat substrate.

**Figure 3:**
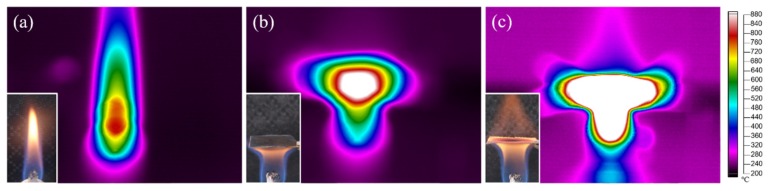
Photos of alcohol flames and temperature field measured by infrared thermal imager: (**a**) uncovered; (**b**) covered by the non-porous metal sheet; (**c**) covered by porous metal wire mesh.

The gas flow velocity simulations revealed some interesting results (Figure 2). The geometric center of the bottom surface of the non-porous metal substrate, where it was struck by the flame volume, had the highest pressure, and the gas pressure decreased from the center of the bottom surface, bypassing its edge, to the top surface of the substrate (Figure 2c). This significant gradient of gas pressure resulted in a gas flow across the surface. A clear and discontinuously flowing gas was observed directly above and below the non-porous metal substrate, and the higher gas velocities were at the edge of the substrate (Figure 2d). In contrast, the gas pressures above and below the porous substrate were almost uniform and there was a significant pressure difference between these two regions. Additional negative and positive pressure regions existed, respectively, on the upper and lower surfaces of a single metal wire (Figure 2g). The gas flows showed a significant improvement in their continuity and uniformity, and their velocities were increased slightly when they passed through the porous substrate (Figure 2h). These simulation results were intended to provide a prediction model of the feeding carbon molecules for MWCNT growth. In addition, the flame covered by porous metal wire mesh exhibited a relatively larger and more uniform temperature field in comparison with the metal sheet (Figure 3). When the alcohol flames were covered by a piece of metal sheet or porous metal wire mesh, the absolute temperature and the high-temperature region were both increased. The alcohol flame covered by the porous metal wire mesh exhibited a relatively wider and more uniform temperature field, and thus this temperature field was more beneficial for large-scale MWCNT growth.

**Figure 4:**
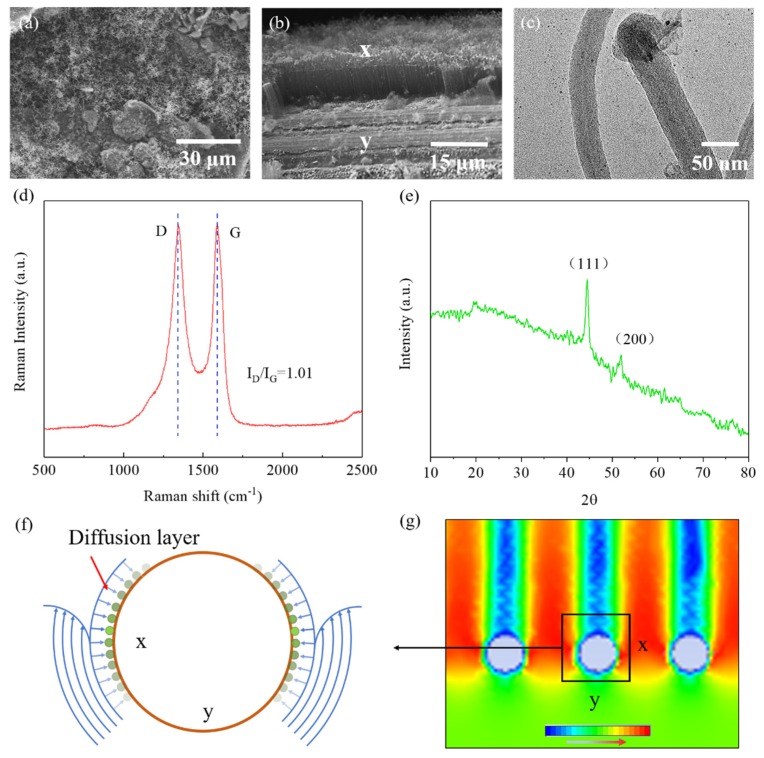
Scanning electron microscope (SEM) images of the CNTs grown on (**a**) metal sheet substrate and (**b**) metal mesh substrate; (**c**) transmission electron microscope (TEM) image and (**d**) Raman spectra of the flame-grown multi-walled carbon nanotubes (MWCNTs); (**e**) X-ray diffraction (XRD) pattern of the as-synthesized sample of MWCNTs; (**f**) diffusion model and (**g**) simulation results of carbon source gas through metal mesh substrate.

As shown in Figure 4a,b, the CNT growth appeared a disorderly tangle on the non-porous substrate, and a vertical CNT array was observed on the porous substrate. The diameter of the flame-grown CNTs (Figure 4c) indicated they were of multi-wall structure. Figure 4d is a Raman spectrum of MWCNTs with a growth time of 10 min. The D peak was caused by the Raman inactive respiratory vibration mode A1g, which indicates defects in the graphite structure (unclosed ports, amorphous carbon, etc.). The G peak was produced by two E2gLaman active vibration modes, indicating an ordered graphite structure. The intensity ratio of the D and G bands (I_D_/I_G_) can be used to evaluate the structural variation of carbon materials in ordered and disordered crystal structures [40]. The intensity ratio of the D band and G band (I(D)/I(G)) was about 1, indicating that the as-synthesized MWCNT contained a higher density of defects in the structure. The disappearance of the radial breathing mode (RBM) confirmed again that no single-walled carbon nanotubes (SWCNTs) were produced. The positions and relative intensities of all the characteristic diffraction peaks (2θ = 44.5° and 51.8°) can be ascribed to the (111) and (200) planes of Ni (JCPDS no. 04-0850) [41] from the XRD pattern of the as-synthesized sample of MWCNTs (Figure 4e). In addition, no diffraction peaks of carbon were observed in the XRD pattern, indicating that the carbon may have existed mainly in an amorphous state.

The growth height of the MWCNT forests changed with different surface zones of the SS wire (Figure 4b). Highly oriented MWCNT forests at the X zone grew regularly in directions perpendicular to the SS wire surface, while the Y zone was bare and not covered by MWCNT forests. These phenomena can be illuminated by the phenomenological model shown in Figure 4e. When the gas flow passed through the porous metal substrate, it split into two layers, the viscous flow layer and the laminar flow layer. The gas in the viscous flow layer that adhered to the wire surface barely flowed, and the gas in the external laminar flow layer contacted the catalyst particles only through radial diffusion, and the diffusion rate was determined by the concentration gradient [42,43,44]. The simulated flow rate cloud diagram (Figure 4f) confirmed the existence of the thin viscous flow layer (blue circle) closely surrounding the wire surface. The X zone exhibited the biggest concentration gradient of carbon source gas from the laminar flow layer to the wire surface, and thus the surrounding surface region is was more favorable for the CNT growth. The gas flow velocity at the Y zone reached the minimum, and the concentration of carbon molecules in this area was the lowest, so there almost no or only a small number of MWCNTs were able to grow.

### 3.2. The Effect of Carbon Source Gas Flow Velocity on CNT Growth Height

**Figure 5:**
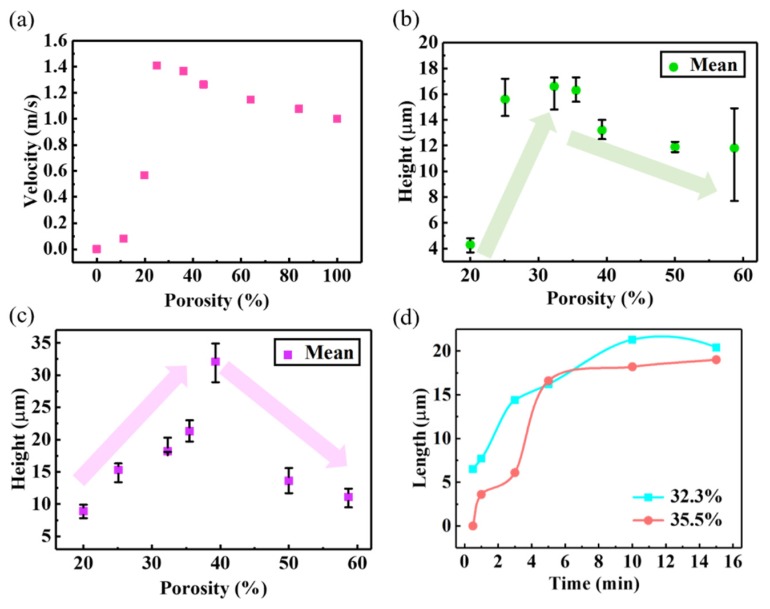
(**a**) Simulation of gas flow rates passing through the metal meshes with different porosities; variation of the MWCNT growth height with the porosity of the metal mesh substrate for growth times of (**b**) 5 min and (**c**) 10 min; (**d**) dependence of MWCNT height on the growth time along with the substrate porosity of 32.3% and 35.5%.

The flow velocity of carbon source gas through a porous substrate plays a key role in the growth height of MWCNT forests, because it determines the feeding amount of the carbon-based molecules to the catalysts on the surface of the porous substrate. The simulation results (Figure 5a, shown in Figure S1) showed that the final output flowing velocity reached the maximum when the substrate porosity was equal to approximately 30%. The growth experiments of MWCNT forests were performed on porous metal substrates, and the porosity of the substrates showed a strong influence on the growth of the MWCNT forests, as shown in Figure 5b,c (shown in Figure S2). When the *t*_g_s was 5 min and 10 min, the maximum heights of MWCNT forests were observed in the porosity range of 25% to 40%, respectively (Figure 5b,c). These observations were consistent with the predicted result of the maximum flow velocity (Figure 5a). Under the same porosity, the as-grown MWCNT forests for 10 min were somehow higher than those grown for 5 min, and the time at which the MWCNT forests stopped growing depended on the porosity of the porous metal substrate (Figure 5d). The *t*_g_ of MWCNTs was determined by the catalyst life [45]. Therefore, the porosity of the porous substrate had an influence on the catalyst life due to the change of feeding flow rate.

### 3.3. Singular Morphology and Growth Mechanism

**Figure 6:**
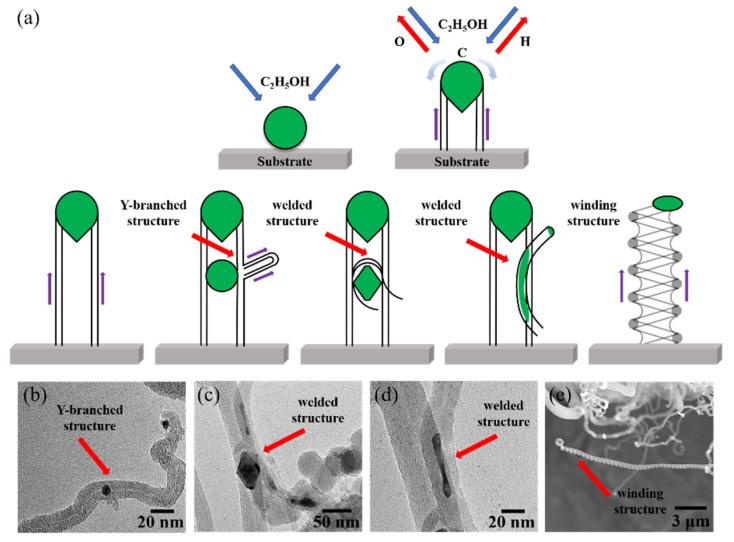
(**a**) Schematic diagrams of growth models; (**b**) TEM of Y-branched structure of flame-grown MWCNTs; (**c**) TEM of top welded structure of flame-grown MWCNTs; (**d**) TEM of middle welded structure of flame-grown MWCNTs; (**e**) TEM of winding structure flame-grown MWCNTs.

Obviously, the growth mechanism of MWCNT forests under consideration follows the “tip growth mode” proposed by Baker and colleagues [46]. Most CNTs are independent and vertical. Bizarrely, some singular CNT structures, such as branch structures, welded structures, and spiral structures, were found when the CNTs grew under an external electric field, which was proven to be capable of changing the external and internal morphology of the MWCNTs [34]. Interestingly, these singular structures were also observed in the ethanol flame synthetic process of CNT forests on the porous substrate. Their production presumably originated from the fragmentation of the catalytic particles, as depicted in Figure 6a. The growth environment of MWCNT forests in flame is extremely bad, and the catalytic particles work in the form of metal droplets at high temperatures. The primary catalyst droplets held aloft by the growing CNTs could be split into smaller catalytic fragments under the gravitational effect and capillary effect in combination with the large internal and external temperature difference of the catalyst droplets. Subsequently, the small split catalyst particles may provide new active sites from their side or upper surfaces where the carbon molecules could be extruded to form small protrusions and ultimately evolve into Y-branched and multi-branched MWCNTs (Figure 6b) [30]. When two MWCNTs contact coincidentally at the catalytic sites, the molten catalyst droplet will weld two MWCNTs together. This part of the catalyst droplet is held at the welding site, and the other part of the catalyst droplet is pushed up with the growth of MWCNTs (Figure 6c,d). The active site region of some Ni catalyst particles is of a nanoscale non-uniform temperature field, resulting in different carbon extrusion rates, and thus will produce enveloping/bending/winding structures of the MWCNTs (Figure 6e). The growth mechanism is similar to Amelinckx’s analysis of CNT coil formation [47,48].

### 3.4. Enhanced Welding Joint Strength of Thermoplastic Composites by MWCNT Forests

**Figure 7:**
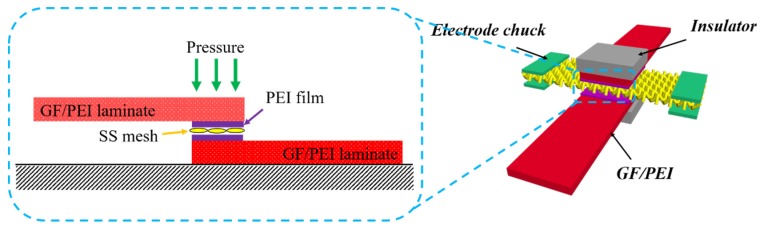
Schematic diagram of the resistance welding of the composite joints with MWCNTs.

**Figure 8:**
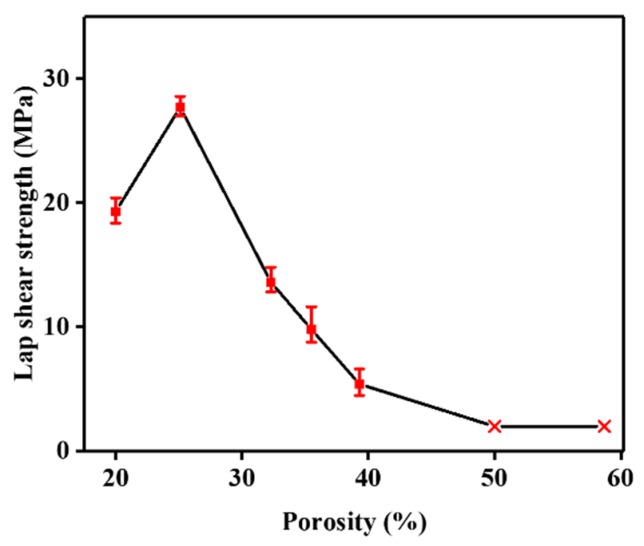
Lap-shear strength (LSS) of the welded joints using bare SS mesh with different porosities as the heating element with a *t*_w_ of 120 s.

The SS mesh is necessary for the resistance welding of thermoplastic composites as a heating element. However, in-plane, non-uniform heat transfer and weak interfacial bonding between SS wire and thermoplastic resin results in weak resistance-welded joints. The surface modification of the SS wires by in situ grafting MWCNTs may be an effective strategy by which to overcome these problems and further improve welded joint strength. Dubé et al. [49] studied the effect of metal mesh size on the heat transfer during the welding process and believed that the porosity of the SS mesh was an important influencing factor for the bonding strength of welded joints. Figure 7 shows the resistance welding process using the MWCNT-forest-decorated SS meshes as heating elements; the bare SS meshes had a porosity of 25.1%, which is the optimum value for the welding of GF/PEI composites (Figure 8). With a metal mesh with a porosity of less than 25%, the molten PEI resin with its high viscosity cannot sufficiently pass through the SS mesh, and thus the welded joint strength is relatively lower. When the porosity is above 25% and continues to increase, the temperature gap between the surface of the SS wire and the center of the SS mesh is increased, the synchronous melting of the thermoplastic resin becomes worse, and the welded joint strength is continuously decreased. Therefore, a metal mesh with a porosity of 25%, which was decorated by MWCNT forests, was used for the resistance welding of thermoplastic composites. 

**Figure 9:**
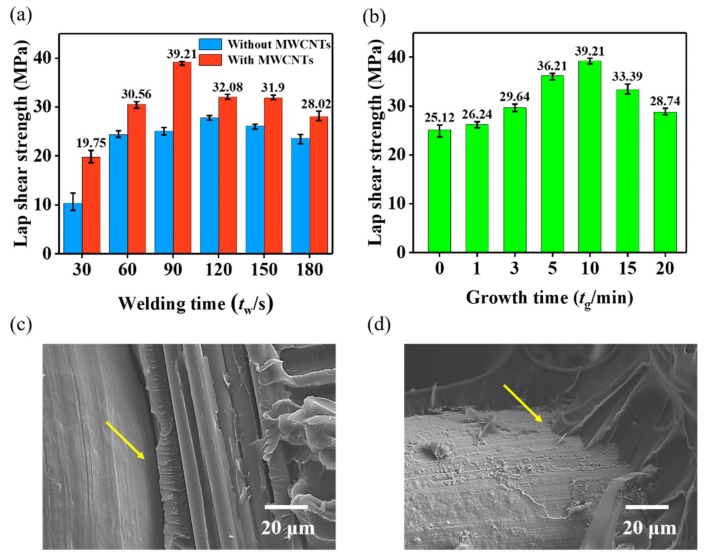
(**a**) LSS comparison of the welded joints without and with MWCNT forests grown for 10 min at different *t*_w_s; (**b**) LSS dependence of the welded joints with MWCNTs on the *t*_g_s at the same *t*_w_ of 90 s; Fracture morphologies of the resin matrix and SS wire (**c**) without and (**d**) with MWCNT forests.

The tensile lap-shear strength (LSS) of the welded joint exhibited a strong dependence on the welding time (*t*_w_) and the *t*_g_ of MWCNT forests (Figure 9a,b). Every LSS value of the welded joints with MWCNTs was obviously higher than that of their counterparts for the same *t*_w_. The maximum LSS was observed when the *t*_g_ reached 10 min, when the height of MWCNT forests was about 15 μm (shown in Figure S2d). The MWCNT forests in the interfacial region acted as similar intermediate connectors to effectively penetrate into the interior of the resin matrix from the heating element surface. The fracture morphologies verified that the debonding surface of the bare SS wire was smooth (Figure 9c); conversely, that of the SS wire decorated by MWCNT forests was tightly bonded and covered by PEI resin (Figure 9d), indicting an enhanced interfacial bonding strength.

**Figure 10:**
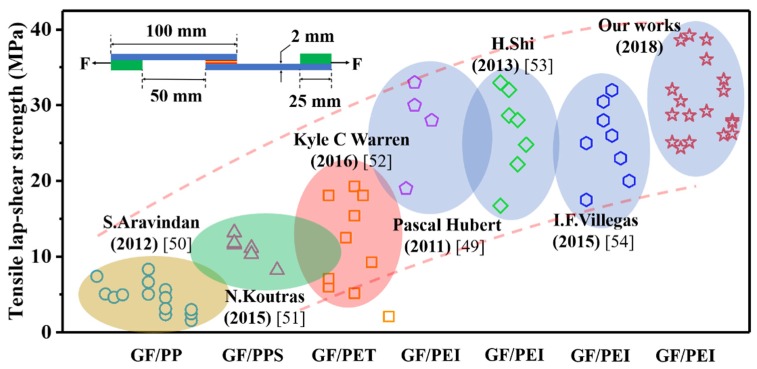
Comparison of welding strength of GF-reinforced thermoplastic composites [49,50,51,52,53,54].

Using MWCNT-forest-decorated SS mesh with a *t*_g_ of 10 min as the heating element made the welded joint reach the maximum LSS of 39.21 MPa with a cost of only 90 s of *t*_w_. Compared with the bare SS mesh, the maximum LSS of the welded joint based on the MWCNT-forest-decorated SS mesh was increased by 41% and the optimum *t*_w_ was shortened by 30 s (Figure 9b). The reported LSS values of welded joints of GF/PEI and other thermoplastic composites are compiled in Figure 10, along with these of the welded joints with MWCNT forests for different *t*_g_ and *t*_w_. Obviously, our work has reached the maximum LSS value of the GF/PEI laminate welding experiments so far. These results demonstrated that the in situ grafting of MWCNT forests onto the surface of SS wires from flame is a significant and easy method for the improvement of thermoplastic composite welded joints. 

## 4. Conclusions

In this study, MWCNT forests were efficiently synthesized from ethanol flame using a porous SS mesh as substrate. The results showed that the growth of MWCNT forests was highly dependent on the initial porosity of the SS mesh substrate and the growth time (*t*_g_). The optimal porosity and *t*_g_ were about 30–40% and 10 min, respectively. Computational fluid dynamics were used to simulate the dependence of the gas flow velocity on the porosity of the SS mesh to elucidate the best growing environment for MWCNT forests. The singular morphologies and growth mechanism of various MWCNTs from ethanol flame were also investigated. The application of the MWCNT-forest-decorated SS meshes as heating elements for the resistance welding of thermoplastic composites could observably improve the welded joint strength, due to the increased interfacial bonding between the SS wire and the resin matrix. The maximum LSS of the welded joints with MWCNT forests reached 39.21 MPa, which was the highest LSS value of GF/PEI welded joints that has been reported in recent years.

## Figures and Tables

**Table 1:** Specifications of the stainless steel (SS) meshes in this work.

Porosity (%)	Wire Diameter (μm)	Aperture (μm)	Number of Meshes
20.00	0.03	0.03	500
25.10	0.10	0.16	100
32.30	0.10	0.22	70
35.50	0.17	0.35	50
39.30	0.25	0.60	30
50.00	0.15	0.50	40
58.70	0.12	0.45	50

## Supplementary Materials

The following are available online at https://www.mdpi.com/2079-4991/9/9/1188/s1, Figure S1: Simulation results of flow velocity for 1 m/s gas flow through porous substrates with different porosities: (a) 0%, (b) 11.1%, (c) 19.8%, (d) 25%, (e) 36%, (f) 44.4%, (g) 64%, (h) 84%, Figure S2: Growth height of MWCNT forests on the substrates with different porosities when *t*_g_ is 10 min: (a) 0%, (b) 11.1%, (c) 19.8%, (d) 25%, (e) 36%, (f) 44.4%, (g) 64%, (h) 84%.
