# Supplementary material for: Flame-Synthesis of Carbon Nanotube Forests on Metal Mesh Structure: Dependence, Morphology, and Application
Source: Nanomaterials (Basel). 2019 Aug 22;9(9):1188. doi: 10.3390/nano9091188 (PMC6780117; doi:10.3390/nano9091188)
Supplement: Supplementary file 1 [file nanomaterials-09-01188-s001.pdf]

Supplementary Information:

Flame-synthesis of Carbon Nanotubes Forests on Metal Mesh Structure: Dependence, Morphology and Application

Xuhai Xiong[a,b*], Pu Zhao[a], Rong Ren[a], Xu Cui[a] and Shude Ji[a]

[a]*Liaoning Key Laboratory of Advanced Polymer Matrix Composites, Shenyang Aerospace University, Shenyang 110136, China*

[b]*Shenyang National Laboratory for Materials Science, Advanced Carbon Division, Institute of Metal Research, Chinese Academy of Sciences, Shenyang 110016, China*

xiongxuhai@126.com (X.X.); 13940086963@163.com (P.Z.); 13630793@qq.com (R.R.); cuixu@sau.edu.cn (X.C.); superjsd@163.com (S.J.)

* Correspondence: xiongxuhai@126.com; Tel.: +86 24 89723970.

Content:

Figure S1. Simulation results of flow velocity for 1m/s gas flow through porous substrates with different porosities: (a) 0%, (b) 11.1%, (c) 19.8%, (d) 25%, (e) 36%, (f) 44.4%, (g) 64%, (h) 84%.

Figure S2. Growth height of MWCNT forests on the substrates with different porosities when t_g is 10 minutes: (a) 0%, (b) 11.1%, (c) 19.8%, (d) 25%, (e) 36%, (f) 44.4%, (g) 64%, (h) 84%.

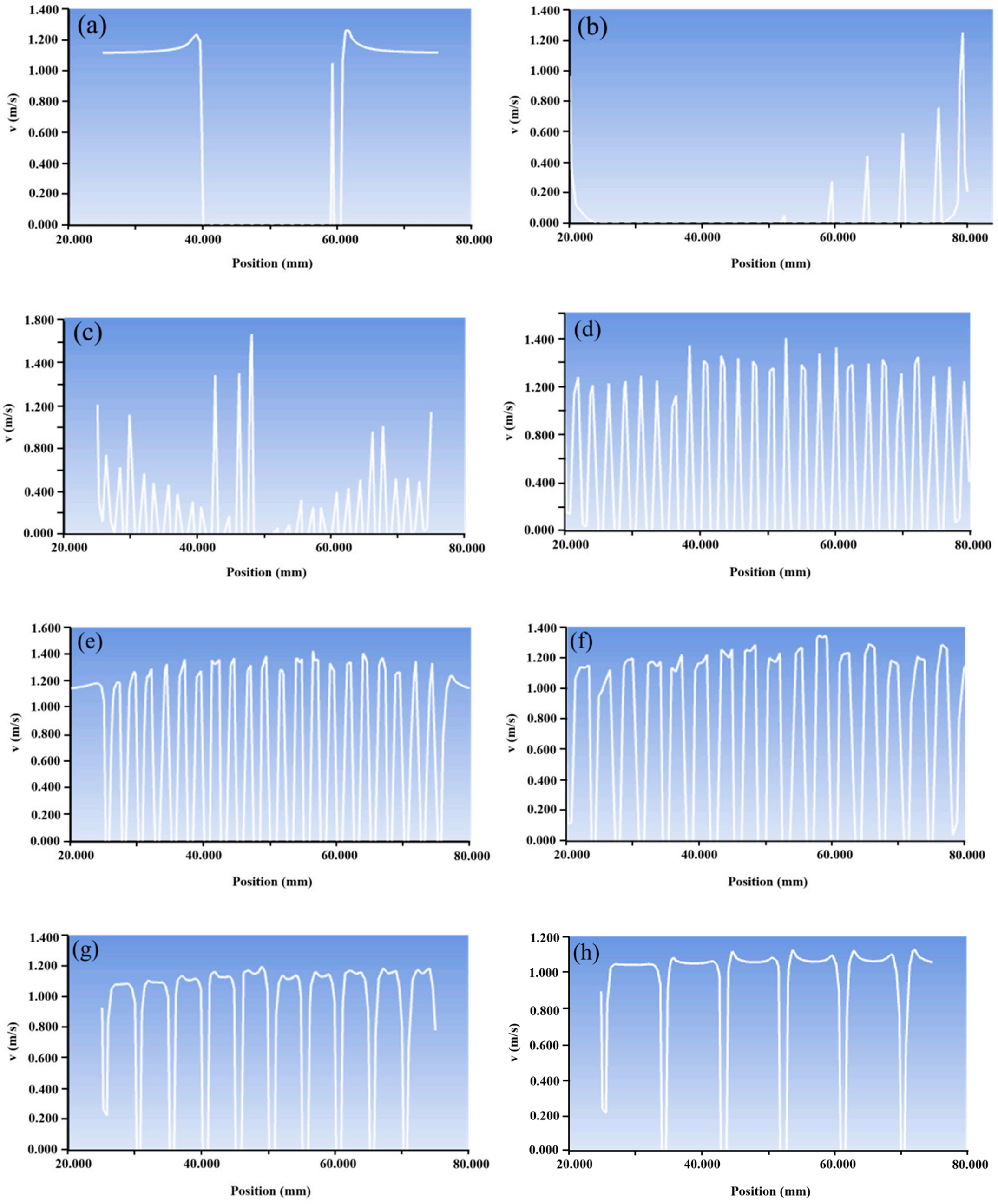

Figure S1. Simulation results of flow velocity for 1m/s gas flow through porous substrates with different porosities: (a) 0%, (b) 11.1%, (c) 19.8%, (d) 25%, (e) 36%, (f) 44.4%, (g) 64%, (h) 84%.

Figure S1 shows the changes in flow velocity for 1m/s gas flow through the porous mesh with different porosities. When the substrate is a non-porous metal sheet, the gas flow cannot go right through it and only passes by its edge with the maximum velocity of about 1.25m/s (**Figure S1a**). The gas flow velocity in the region covered by a porous mesh with a porosity of 11.1% is zero due to the strong blocking effect (**Figure S1b**).

As the porosity of the substrates is increased to 19.8%, the gas flow can pass through the porous mesh at a very low velocity and form an unsteady nonuniform flow field (**Figure S1c**). When the porosity of the substrates is between 25% and 44%, all substrates can produce uniform flow fields together with high flow velocities (**Figures S1d-f)**. The velocities of the gas flow through these substrates reach the maximum value of about 1.4m/s due to the optimum additional pressure generated from the restriction of metal mesh. As the porosity of the substrates continues to increase, the additional pressure decreases and thus the gas flow velocity gradually falls to about 1m/s (**Figures S1g-h**). The flame volume is the essential materials for the flame-growth of CNT forest. Therefore, the flow velocity of the gas flame plays an important role in the quality of CNT growth.

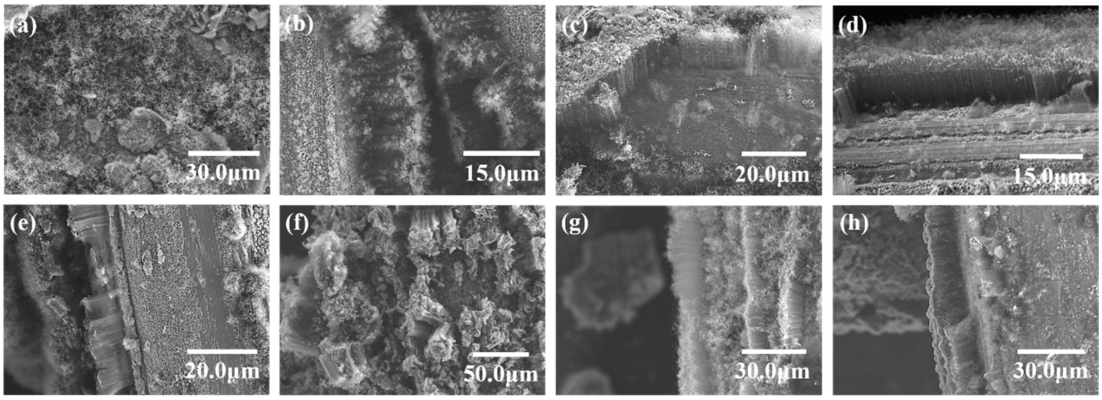

Figure S2. Growth height of MWCNT forests on the substrates with different porosities when t_g is 10 minutes: (a) 0%, (b) 11.1%, (c) 19.8%, (d) 25%, (e) 36%, (f) 44.4%, (g) 64%, (h) 84%.
